# Mr. Bok’s “Attack”

**Published:** 1908-04

**Authors:** 


### Mr. Bok's "Attack"

AT a recent meeting of the Philadelphia branch of the American Pharmaceutical Association, Mr. Edward W. Bok, editor of the *Ladies' Home Journal,* made an address in the course of which he said that a large number of physicians were prescribing "nostrums." Since that address, Mr. Bok has been the recipient of a number of letters and several pleasant reprints, editorial and otherwise, taking him to task for "attacking" the medical profession.

As a matter of fact Mr. Bok did not attack the medical profession at all. His address was not an attack; it was a simple statement of facts based upon his own obervations and not the result of hearsay or the late reading of circular correspondence.

In the issue of the *Journal of the American Medical Association of* March 21, Mr. Bok has a communication which fairly and rather effectually answers the criticisms which have been gratuitously handed him. There probably was not any more animus in these criticisms than there was in the alleged "attack" which Mr. Bok is supposed to have made on the profession; but the reply which he has written is interesting. He sharply defines his position and tells what a "nostrum" is. More than that he gives specific instances where he has personal knowledge of the prescribing of what he designates as "nostrums," and cites one instance where a well-known teacher advised his students to use the very preparations which the Council of Pharmacy and Chemistry most strenuously denounce. Mr. Bok's reply is good reading, and quite definitely shows that his "attack" was not anything in the nature of an attack at all, but an address of considerable interest.

